# Laparoscopic complete mesocolic excision with central vascular ligation for splenic flexure colon cancer: short- and long-term outcomes

**DOI:** 10.1007/s00464-021-08559-y

**Published:** 2021-05-24

**Authors:** Kazuki Ueda, Koji Daito, Hokuto Ushijima, Yoshinori Yane, Yasumasa Yoshioka, Tadao Tokoro, Masayoshi Iwamoto, Toshiaki Wada, Yusuke Makutani, Junichiro Kawamura

**Affiliations:** Department of Surgery, Kindai University Faculty of Medicine, 377-2, Ohnohigashi, Osaka Sayama, Osaka, 589-8511 Japan

**Keywords:** Colorectal surgery, Laparoscopic surgery, Splenic flexure, Surgical outcome, Transverse colon cancer, Descending colon cancer

## Abstract

**Background:**

Complete mesocolic excision (CME) with central vascular ligation (CVL) for colon cancer is an essential procedure for improved oncologic outcomes after surgery. Laparoscopic surgery for splenic flexure colon cancer was recently adopted due to a greater understanding of surgical anatomy and improvements in surgical techniques and innovative surgical devices.

**Methods:**

We retrospectively analyzed the data of patients with splenic flexure colon cancer who underwent laparoscopic CME with CVL at our institution between January 2005 and December 2017.

**Results:**

Forty-five patients (4.8%) were enrolled in this study. Laparoscopic CME with CVL was successfully performed in all patients. The median operative time was 178 min, and the median estimated blood loss was 20 g. Perioperative complications developed in 6 patients (13.3%). The median postoperative hospital stay was 9 days. According to the pathological report, the median number of harvested lymph nodes was 15, and lymph node metastasis developed in 14 patients (31.1%). No metastasis was observed at the root of the middle colic artery or the inferior mesenteric artery. The median follow-up period was 49 months. The cumulative 5-year overall survival and disease-free survival rates were 85.9% and 84.7%, respectively. The cancer-specific survival rate in stage I-III patients was 92.7%. Recurrence was observed in 5 patients (11.1%), including three patients with peritoneal dissemination and two patients with distant metastasis.

**Conclusions:**

Laparoscopic CME with CVL for splenic flexure colon cancer appears to be oncologically safe and feasible based on the short- and long-term outcomes in our study. However, it is careful to introduce this procedure to necessitate the anatomical understandings and surgeon’s skill. The appropriate indications must be established with more case registries because our experience is limited.

The safety and benefits of laparoscopic surgery for colorectal cancer have been demonstrated and confirmed by several randomized controlled trials (RCTs) and meta-analyses [1–4]. In Japan, the Japan Clinical Oncology Group (JCOG) 0404 also conducted an RCT to establish the noninferiority of laparoscopic surgery to open D3 surgery for stage II or III colon cancer [5]. The short-term outcomes were the safety and clinical benefits of laparoscopic surgery, as shown in a previous RCT [6]. Concerning survival outcomes, patients who underwent laparoscopic surgery had a relatively high overall survival rate, which was better than expected [7]. Since its equivalence to open surgery has become apparent, laparoscopic surgery has been widely accepted as a standard strategy for colorectal malignancies.

Complete mesocolic excision (CME) with central vascular ligation (CVL) for colon cancer was introduced by Hohenberger and has improved oncologic outcomes after surgery [8–10]. This concept of CME with CVL for colon cancer is to extract the tumor in its entirety, with free margins of the bowel and mesocolon, and including lymphatic spread along the supplying vessels at its origin; this concept is similar to that of total mesorectal excision (TME) for rectal cancer described by Heald et al. [11]. Although the extent of longitudinal resection differs in Europe and Japan and is still debatable, the Japanese surgeons follow the concept that is known as D3 lymph node dissection, advocated by the Japanese Society for Cancer of the Colon and Rectum (JSCCR) guidelines [12, 13].

Transverse colon and descending colon cancers were excluded from the majority of previous prospective studies because of complex anatomy, technical difficulties, and difficulty in judging the related central vessels and regional lymph nodes. Since several decades have passed since the first described laparoscopic colectomy [14], with a better understanding of surgical anatomy and improvements in laparoscopic surgical techniques and innovative surgical devices, laparoscopic surgery for splenic flexure colon cancer was recently adopted [15, 16].

This retrospective study aimed to evaluate the short- and long-term outcomes of laparoscopic resection for splenic flexure colon cancer in our single institution.

## Materials and methods

### Patients

We retrospectively analyzed a total of 938 patients who underwent laparoscopic colectomy for colorectal cancer at Kindai University Hospital between January 2005 and December 2017 from our prospectively collected database. Splenic flexure colon cancer was defined as a tumor located between the distal third of the transverse colon and the first proximal portion of the descending colon within 10 cm of the splenic flexure. During the study periods, 45 consecutive patients (4.8%) who underwent laparoscopic curative resection for pathologically proven primary colon adenocarcinoma were reviewed retrospectively. Our exclusion criteria for laparoscopic surgery were as follows: bulky tumor invasion to adjacent organs (stage T4b), apparent lymph node metastasis in the root of the inferior mesenteric artery or para-aorta, emergency surgery for bowel obstruction and/or perforation due to the tumor and synchronous unresectable multiple liver metastases. In patients with altered colon anatomy due to previous colorectal surgery, an open procedure was also adopted.

All the patients underwent preoperative total colonoscopy and plane- or contrast-enhanced computed tomography (CT) to determine the exact tumor location and to detect distant metastases. The feeding artery to the tumor was also confirmed by three-dimensional (3D) CT imaging when possible. Histology was confirmed by endoscopic biopsy prior to surgery for all patients.

The disease stage was evaluated according to the American Joint Committee on Cancer Staging, 8^th^ Edition.

This study was approved by the Institutional Review Board of the Kindai University Faculty of Medicine. Due to the retrospective nature of the study, written informed consent was not obtained. We used the opt-out approach to disclose the study information.

### Surgical procedure

Oncological surgical resection was performed as described in the JSCCR guidelines [12]. All laparoscopic surgeries were performed by surgeons who passed the skill accreditation system for laparoscopic gastrointestinal surgery, established by the Japanese Society for Endoscopic Surgery (JSES). All cases were performed only by three highly skilled laparoscopic colorectal surgeons.

Under general anesthesia, the patient was placed in the supine position in the modified lithotomy position using stirrups. Both arms were alongside the body. The first camera trocar (12 mm) was inserted at 3.5 cm of the umbilical incision via a round-shaped E·Z ACCESS with LAP PROTECTOR MINI device (Hakko Co., Ltd., Tokyo, Japan). Pneumoperitoneum was established (up to 8 mmHg) with carbon dioxide through the camera trocar. Four additional 5-mm trocars were inserted in an inverted trapezoid shape under direct vision around the umbilical lesion. The omentum and transverse colon were moved toward the upper abdomen. A medial-to-lateral approach was utilized for this procedure. CME was performed with careful mobilization of colonic mesentery avoiding mesenteric injury, and CVL was performed following a preoperative CT scan with regard to the correlation between the tumor and supplying/drainage vessels. In the case of T3 or T4 tumors, partial omentectomy around the tumor was performed. The transverse mesentery was dissected at the lower border of the pancreas. The splenic flexure was fully mobilized with a securely preserved pancreas and spleen before anastomosis. The tumor-bearing segment was extracted through umbilical mini-laparotomy. The reconstruction was made by functional end-to-end anastomosis (FEEA), the triangulating stapling technique (TST), or the double stapling technique (DST). The procedure of FEEA was as follows: the bowel was fully mobilized, and the mesentery was divided at the proposed lines of resection. The antimesenteric borders of both bowels were sutured by a single suture. Then, 1-cm enterotomies were made within the segment to be resected 1 cm beyond the site of vascular division. A side-to-side anastomosis was made using the linear cutting stapler (proximate® linear cutter, TLC75; Ethicon Endosurgery, Cincinnati, OH, USA). After the linear cutting stapler was withdrawn, both bowel ends were transected just beyond the enterotomy with the linear cutting stapler [17, 18]. The procedure of TST was also as follows: Three times of the TA™ 55 (Medtronoc, Minneapolis, MN, USA) was used to staple the approximated bowel ends. This technique was an alternative procedure performed by the hand-sewn technique. The important thing is that each stapled line was overlapped to reinforce the anastomosis [19, 20]. The procedure of DST was performed the conventional fashion same as sigmoidectomy or anterior resection.

### Surveillance after surgery

All patients were followed under the JSCCR guidelines 2019 [12]. As described in the guidelines, all patients were followed up for 5 years after surgery. The patients were observed regularly by surgeons every 3 months for the first 3 years and then every 4–6 months for the next 2 years. The physical examinations and blood tests were done at each visit. Chest and abdominal CT scans were done every 6 months. A colonoscopy was done every 2 years. If metastasis or recurrence was suspected, more detailed diagnostic examinations were performed.

### Measured outcomes

The following variables were measured: patient demographics, intra- and postoperative data, and complications according to the Clavien-Dindo classification [21]. Overall survival, cancer-specific survival, rate of recurrence, and site of recurrence were also analyzed during the follow-up period.

### Statistical analysis

Data are presented as the median and range based on their distribution. Survival analysis was performed using the Kaplan–Meier method. JMP Pro software ver. 15 (SAS Institute Inc., Cary, NC, USA) was used for statistical analysis.

## Results

During the study period, 45 consecutive patients (4.8%) who underwent laparoscopic curative resection for pathologically proven splenic colon cancer were analyzed. There were 20 men and 25 women ranging in age from 39 to 89 years, with a median age of 71 years. The tumor location included the transverse colon in 31 patients and the descending colon in 14 patients. The patient demographics are shown in Table 1.

**Table 1:** Patients’ preoperative characteristics

	All patients (n = 45)	
Age (median [range], y.o.)	71	[39–89]
Gender (Male/Female) (no.)	20/25	
BMI (median [range], kg/m^2^)	21.8	[15.8–29.1]
Tumor location (T/D, no.)	31/14	
ASA classification (no.)		
1	2	
2	33	
3	10	
4	0	
Stage (no., (%))		
0	1	(2.2)
I	9	(20.0)
II	20	(44.4)
III	13	(28.9)
IV	2	(4.4)

*BMI* body mass index, *T* transverse colon, *D* descending colon, *ASA*, the American Society of Anesthesiologists

The median operative time was 178 min (range, 102–433 min.). The median estimated blood loss was 20 g (range, 5–946 g). There were four intraoperative complications: two splenic injuries, one pancreatic injury, and one bowel injury. A soft coagulation system was used to resolve bleeding from the splenic injuries. However, pancreatic injury was necessitated distal pancreatectomy without requiring conversion to an open procedure. Two patients (4.4%) required conversion: one for bowel injury and one for the difficulty of visibility due to severe obesity. All patients successfully underwent CME with CVL. Bowel reconstruction was performed for 34 patients via FEEA, 10 patients via the TST, and one patient via the DST. The postoperative recovery course of patients, including the median time to ambulation, flatus, and soft diet intake, was one day (range, 1–3 days), two days (range, 0–4 days), and four days (range, 1–10 days), respectively. The median postoperative hospital stay was nine days (range, 6–227 days). Intra- and postoperative outcomes are shown in Table 2.

**Table 2:** Operative and postoperative clinicopathological characteristics

	All patients (n = 45)	
Operative time (median [range], min)	178	[102–433]
Estimated blood loss (median [range], g)	20	[5–946]
Anastomosis		
FEEA/TST/DST (no.)	34/10/1	
Intraoperative complications (no., %)	4	(8.9)
Conversions (no., %)	2	(4.4)
Specimen		
Tumor size (median [range], mm)	35	[5–65]
Tumor differentiation		
Well or moderately differentiated (no., %)	43	(95.6)
Poorly differentiated or mucinous (no., %)	2	(4.4)
Harvested nodes (median [range], no.)	15	[6–56]
Proximal margin (median [range], mm)	80	[30–180]
Distal margin (median [range], mm)	110	[50–400]
Postoperative course		
Ambulation (median [range], days)	1	[1–3]
Flatus (median [range], days)	2	[0–4]
Oral intake (median [range], days)	4	[1–10]
Postoperative length of stay (median [range], days)	9	[6–227]
Postoperative complications (CD ≥ grade 2) (no., %)	6	(13.3)
Anastomotic leakage (no., %)	1	(2.2)
Recurrence (no., %)	5	(11.1)
Follow-up (median [range], months)	48.9	[4–174.8]
Death (no., %)	6	(13.3)

*FEEA* functional end-to-end anastomosis, *TST* triangulating stapling technique, *DST* double stapling technique, *CD* Clavien-Dindo classification

Postoperative complications (Clavien-Dindo classification ≥ grade 2) developed in six patients (13.3%): anastomotic leakage in one, duodenal stenosis in one, paralytic ileus in two, aspiration pneumonia in one, and surgical site infection in one. Major anastomotic leakage developed in one patient who had undergone emergency surgery in which a diverting stoma was temporarily created. The patient necessitated a long-term admission period and died 289 days postoperatively because of septic shock with chronic heart failure. A ventral hernia developed in two patients as a late complication. One of these patients underwent laparoscopic ventral hernia repair 6 months after primary surgery.

**Fig. 1:**
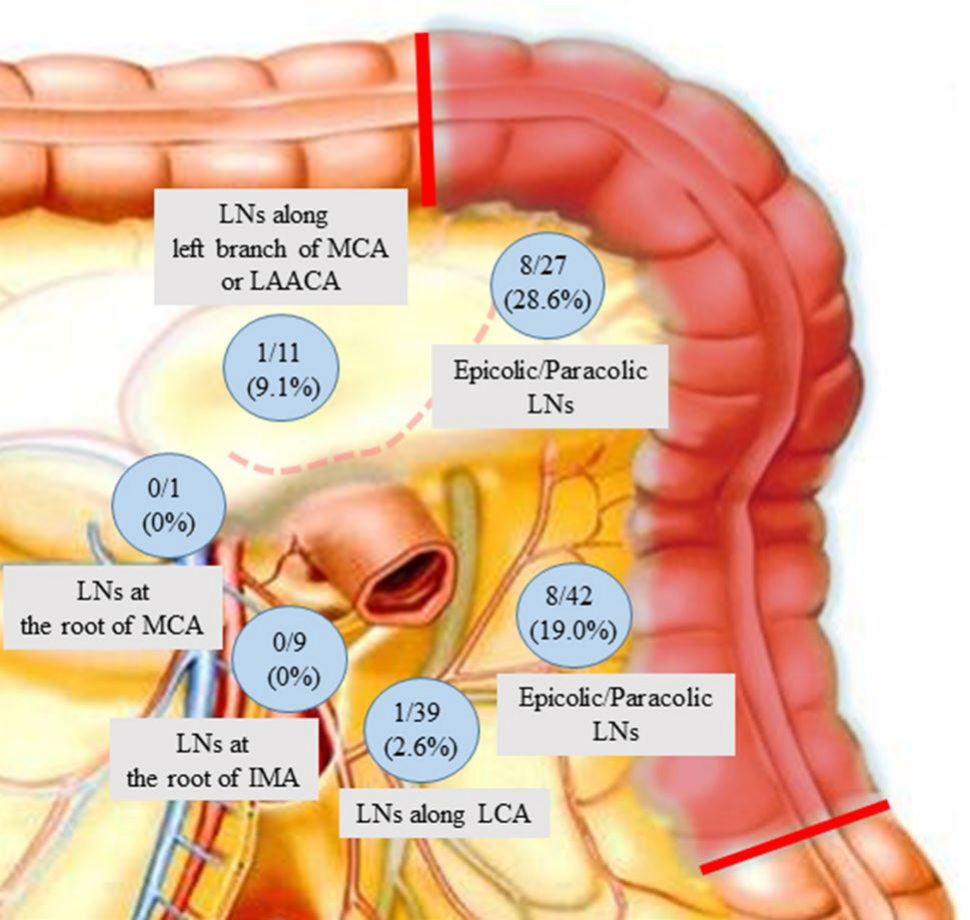
Distribution of lymph node metastasis in 45 patients who underwent curative resection for splenic flexure colon cancer. *LNs* lymph nodes, *MCA* middle colic artery; *LAACA* left accessory aberrant colic artery, *LCA* left colic artery, *IMA* inferior mesenteric artery

The median tumor size was 35 mm (range, 5–65 mm). The median number of harvested lymph nodes was 15 (range, 6–56) (Table 2). Lymph node dissection was performed according to the preoperative 3D-CT images and clinical stage. Lymph nodes located at the root of the middle colic and the inferior artery were dissected in patients with clinical stage II or III disease. Lymph node metastasis developed in 14 (31.1%) of the 45 patients. The distribution of lymph node metastasis is shown in Fig. 1. No metastasis was observed histologically at the root of the middle colic artery or the inferior mesenteric artery.

**Fig. 2:**
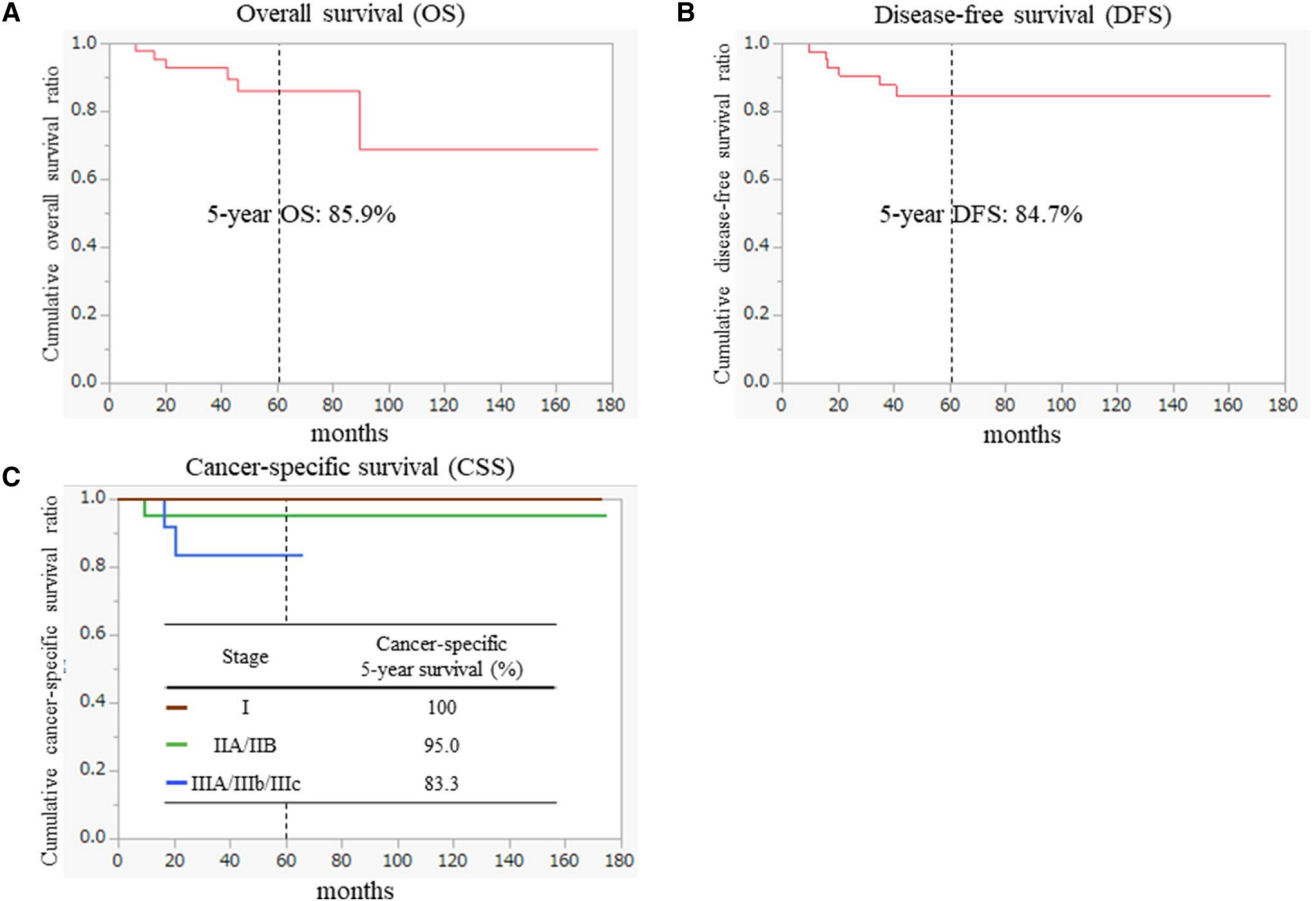
Oncologic long-term outcomes. **A** Overall survival. **B** Disease-free survival. c Cancer-specific survival. The table shown in (**C**) indicates differences in cancer-specific survival according to disease Stage I to III

The median follow-up period was 49 months (range, 4–175 months). The cumulative 5-year overall survival rate in patients with stage I-III disease was 85.9%, and the 5-year disease-free survival rate was 84.7%. The 5-year cancer-specific survival rate in patients with stage I-III disease was 92.7% (Fig. 2). Recurrence was observed in five patients (11.1%). The distributions of patients who experienced recurrence are shown in Table 3. Three of five patients experienced peritoneal dissemination: one patient with stage T4a disease and two patients with stage T3 disease. The other two patients developed distant metastases. Local recurrence was not observed during the follow-up period.

**Table 3:** Clinical characteristics of cancer recurrence among the total patient

Gender	Age	Location	Tumor stage	TNM staging	Previous metastasis	Tumor differentiation	Harvested lymph nodes (no.)	Adjuvant chemotherapy	Disease-free survival (months)	Recurrent site	Outcome
M	73	T	3	IIA	None	Well	15	None	11.2	Liver	Alive
F	76	T	3	IIIB	None	Well	14	None (Refused)	6.7	Peritoneum	Alive
M	69	T	3	IIIB	None	Mode	15	CapeOX	4.9	Peritoneum	Death ^†^^1^
M	56	T	4a	IIIC	None	Muc	32	CapeOX	10.9	Peritoneum	Death ^†2^
M	69	T	4a	IV	Oligometastatic liver	Mode	16	FOLFOX + Bev	28.9	Lung, Liver	Alive

^†^^1^This patient died caused by peritoneal dissemination followed by lung metastases

^†2^This patient died caused by peritoneal dissemination followed by refractory cachexia

*T* Transverse colon, *Well* well differentiated adenocarcinoma, *Mode* Moderately differentiated adenocarcinoma, *Muc* Mucinous adenocarcinoma, *CapeOX* Capecitabine + Oxaliplatin, *FOLFOX* Oxaliplatin + Leucovorin + 5-FU, *Bev* Bevacizumab

## Discussion

Splenic flexure colon cancer located in the distal transverse colon and the proximal descending colon accounts for approximately 2–5% of all colorectal cancers [22–25]. The definition of splenic flexure colon cancer is diverse. In this study, splenic flexure colon cancer was defined as a tumor located between the distal third of the transverse colon and the first proximal portion of the descending colon within 10 cm of the splenic flexure. Following this definition, the incidence of splenic flexure colon cancer in the current study was 4.8% of the patients presenting with all colon cancers in our institution.

In our institution, laparoscopic colorectal surgery was first introduced in 1995, and its indications have been expanded by the technical level of proficiency. After standardizing the procedure, it has been adopted for splenic flexure colon cancer since 2005. The indications for surgical resection margins and lymphadenectomy were based on the JSCCR guidelines. Although the definition of splenic flexure colon cancer and the surgical procedure for splenic colon cancer are diverse, our short-term outcomes were acceptable according to previous reports (Table 4) [26–32].

**Table 4:** Patients’ series of splenic flexure colon cancer or transverse and descending colon cancer in the literature

	Akiyoshi et al. [26]	Nakshima et al. [27]	Kim et al. [28]	Yamaguchi et al. [29]	Watanabe et al. [30]	Toritani et al. [31]	Blacale et al. [32]
Number of patients (no.)	260	33	33	958	31	33	112
Operative time (median or mean [range], min.)	194 [95–405]	209 [144–299]	295 [255–362.5]	–	206 [-]	179 [137–195]	155.2 [–]
Estimated blood loss (median [range], g)	18 [0–740]	15 [0–1150]	–	–	52.5 [–]	30 [10–50]	–
Intraoperative complications (%)	3.5	6.1	9.1	3.2	0	0	3.6
Conversions (%)	0.8	3.0	6.1	4.5	0	0	5.4
Postoperative complications (%)	6.9	6.0	18.2	15.8	9.7	6.1	18.8
Anastomotic leakage (%)	0.8	3.0	0	1.0	0	0	3.6

As in all colon cancer surgeries, a correct CME procedure, which includes sharp dissection along the embryological planes and obtaining a specimen with intact mesocolic fascia, which envelope the lymphatic drainage of the tumor, is mandatory. The tumor around the splenic flexure is dominated by the origin of the inferior mesenteric artery or the middle colic artery. Therefore, a variety of lymphatic drainage events along with the blood supply of the splenic flexure are observed. To determine the resection margin and the area for lymphadenectomy, we applied 3D CT angiography over a routine preoperative workup. Most tumors in the splenic flexure are supplied by the left colic artery originating from the inferior mesenteric artery. Griffiths performed colic vessel aortography on 71 patients. Of these patients, the percentage of blood supply to the splenic flexure from the left colic artery was 66% on aortograms [33]. In recent years, Fukuoka et al. [34] reported the classification of blood supply to the splenic flexure using CT angiography and colonography for 202 patients. They reported that 53.7% of the blood to the splenic flexure was supplied by the left colic artery. In our study, the percentage of blood supplied by the left colic artery was 81.6% (31/38) among patients who underwent CT angiography or contrast-enhanced CT. This ratio was similar to that in the report by Nakashima et al. [27], in which 89% of splenic flexure colon cancers were supplied through the left colic artery according to the operative records. Nakagoe et al. [35] described a similar study on 27 patients with splenic flexure colon cancer who underwent splenic flexure segment resection. They also reported no metastases at the root of the inferior mesenteric artery or the middle colic artery and concluded that the routine extended right hemicolectomy might not be necessary to cure splenic flexure colon cancer. As indicated in Fig. 1, most of the metastatic lymph nodes existed in the epicolic/paracolic area, and no metastatic lymph nodes existed in the lymph nodes at the root of the inferior mesenteric artery or middle colic artery. Moreover, there was no case of lymph node recurrence during the follow-up period. Our indication for the surgical resection margin and lymphadenectomy was appropriate for splenic flexure colon cancer.

**Fig. 3:**
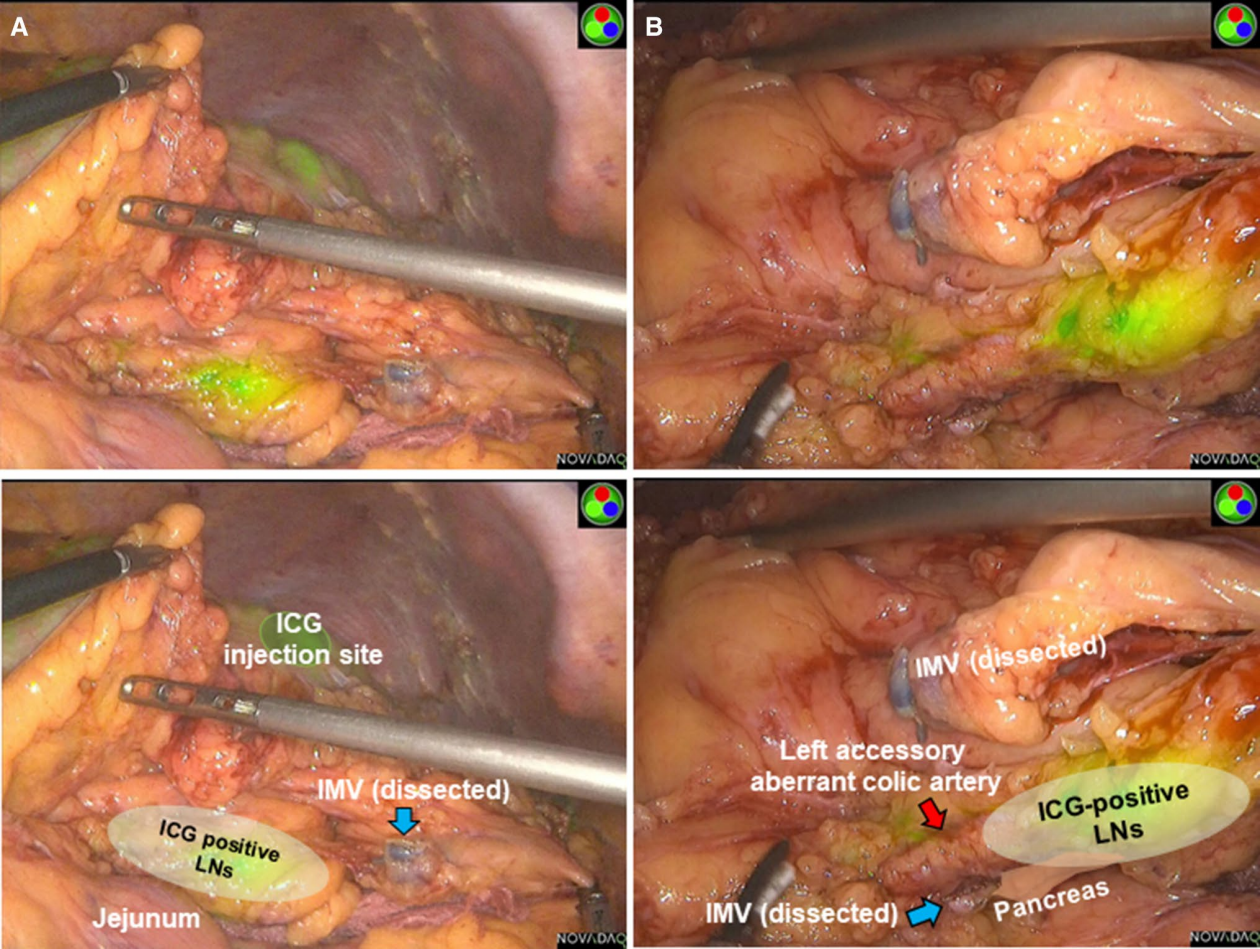
Intraoperative ICG fluorescence imaging. (e.g., ICG-positive lymph nodes identified around the left accessory aberrant artery). **A** Distant view. **B** Close view. The names of the structures are shown in the figures below. *ICG* indocyanine green, *IMV* inferior mesenteric vein, *LNs* lymph nodes

In recent years, various studies have described intraoperative laparoscopic real-time indocyanine green (ICG) fluorescence imaging using a near-infrared camera system to recognize the pathway of lymphatic drainage from the tumor [30, 36, 37]. This useful modality can detect the precise dissection area of the colic mesentery for CME. Although ICG is not a cancer-specific fluorophore, it can potentially detect lymph node metastasis. However, it is limited by the fact that it cannot detect the ICG spreading area, and it may alter the lymphatic flow. Regarding patient-specific limitations, if a patient is obese with a thick, fat mesentery, lymphatic spread cannot be observed during the procedure. Concerning cancer-specific limitations, ICG cannot enter the lymph node sinus in highly metastatic lymph nodes. As a result, ICG cannot be observed, or it may alter lymphatic flow from the tumor. Four of 57 patients with splenic flexure colon cancer who analyzed in our previous prospective study on the intraoperative detection of lymphatic flow and lymph nodes using ICG fluorescence imaging were included also in this study [38]. Three of these four patients who were pathologically diagnosed with stage N1a/b disease had ICG-positive lymph nodes along the left colic artery, middle colic artery, and left accessory aberrant colic artery (Fig. 3). In the other patient with metastatic lymph nodes and liver metastases, lymphatic flow and lymph nodes were not observed. ICG fluorescence imaging seems to be an informative modality to identify potentially metastatic lymph nodes for early colon cancer or clinically node-negative advanced colon cancer. If the cancer-specific agents that can be used to identify metastatic lymph nodes during surgery are developed, it will be possible to perform patient-specific or tumor-specific surgery in the near future.

Regarding the long-term follow-up, the 5-year overall survival and disease-free survival rates in patients with stage I-III disease were 85.9% and 84.7%, respectively. However, more patients died of other diseases than cancer progression. The cancer-specific survival rate in this series was 92.7% (100% in patients with stage I, 95% in patients with stage II, and 83.3% in patients with stage III disease). This result was similar to that reported in patients who underwent laparoscopy in JCOG 0404, a phase 3 RCT [7]. Moreover, Hohenberger et al. [8] were the first to describe CME with CVL for colon cancer surgery. They reported 5-year cancer-related survival rates of 99.1%, 91.4%, and 70.2% in patients with stage I, II and III disease, respectively, 4.4% of whom had splenic flexure colon cancer [8].

During the follow-up period, five patients (11.1%) experienced recurrence, including three with peritoneal dissemination (6.7%). From 2000 to 2020, six retrospective case studies or comparative studies on splenic flexure colon cancer were performed except in the case report or technical report (Table 5). The rate of local recurrences, such as the anastomotic recurrence, was 0.6–4.3%. The rates of lymph node metastasis, peritoneal recurrence, and distant metastasis were 1.8–4.2%, 2.0–20.6%, and 3.6–23.5%, respectively. The patients’ backgrounds (e.g., procedure and operative stage) in these studies were quite different. However, the results of the latter studies (from 2015 to 2020), including laparoscopic case studies, showed a similar incidence of recurrence to that in the present study, though the mechanism of these patterns of recurrence was unclear. To identify the mechanism involved, we should examine a large number of case registries on splenic flexure colon cancer.

**Table 5:** Recurrent cases of splenic flexure colon cancer in the literature

	Nakagoe et al. [39]	Kim et al. [24]	Pisani Ceretti et al. [15]	Kim et al. [28]	Rega et al. [16]	Bracale et al. [32]	Current study
Number of patients	34	167	23	51	57	112	45
Open/Lap (no.)	Open	Open	Lap	Open (18)/Lap (33)	Open (47)/Lap (10)	Lap	Lap
Follow-up (months)	30	82	33	59	42	43	49
Local recurrence (%)	-	1 (0.6)	1 (4.3)	1 (2.0)	2 (3.5)	4 (3.6)	0
Lymph node metastasis (%)	-	7 (4.2)	–	1 (2.0)	–	2 (1.8)	0
Peritoneal dissemination (%)	7 (20.6)	16 (9.6)	–	1 (2.0)	–	5* (4.5)	3 (6.7)
Distant metastasis (%)	8 (23.5)	25 (15.0)	1 (4.3)	3 (5.9)	12 (21.1)	4 (3.6)	2 (4.4)

*Two cases of 5 peritoneal disseminations were observed simultaneously occurred lymph node metastasis and distant metastasis, respectively

The present study had some limitations. First, it was a retrospective study, and the case number was small. Second, this study was historically reviewed; it was not a comparative study with open surgery. Third, some technical errors and biases could not be ignored. For example, we did not estimate the injury of the mesentery, ‘incomplete CME’, in each case.

## Conclusion

In conclusion, laparoscopic CME with CVL for splenic flexure colon cancer appears to be oncologically safe and feasible based on the short- and long-term outcomes reported in our study. However, this procedure is challenging because of the understanding of surgical anatomy and the necessity of surgeon’s skill to balance the avoidance of other organ injury and the curability of cancer. The appropriate indications must be established with more case registries because our experience is limited.
